# Adjustments of plant primary metabolism in the face of climate change

**DOI:** 10.1093/jxb/eraf116

**Published:** 2025-03-11

**Authors:** Mustafa Bulut, Esra Karakas, Alisdair R Fernie

**Affiliations:** Max Planck Institute of Molecular Plant Physiology, Am Mühlenberg 1, 14476 Potsdam-Golm, Germany; Max Planck Institute of Molecular Plant Physiology, Am Mühlenberg 1, 14476 Potsdam-Golm, Germany; Max Planck Institute of Molecular Plant Physiology, Am Mühlenberg 1, 14476 Potsdam-Golm, Germany; Universidade Federal de Viçosa, Brazil

**Keywords:** Abiotic stress, climate change, crop species, primary metabolism

## Abstract

Plant metabolism is profoundly affected by various abiotic stresses. Consequently, plants must reconfigure their metabolic networks to sustain homeostasis while synthesizing compounds that mitigate stress. This aspect, with the current intensified climate impact results in more frequent abiotic stresses on a global scale. Advances in metabolomics and systems biology in past decades have enabled both a comprehensive overview and a detailed analysis of key components involved in the plant metabolic response to abiotic stresses. This review addresses metabolic responses to altered atmospheric CO_2_ and O_3_, water deficit, temperature extremes, light intensity fluctuations including the importance of UV-B, ionic imbalance, and oxidative stress predicted to be caused by climate change, long-term shifts in temperatures, and weather patterns. It also assesses both the commonalities and specificities of metabolic responses to diverse abiotic stresses, drawing on data from the literature. Classical stress-related metabolites such as proline, and polyamines are revisited, with an emphasis on the critical role of branched-chain amino acid metabolism under stress conditions. Finally, where possible, mechanistic insights into the regulation of metabolic processes and further outlook on combinatory stresses are discussed.

## Teaser

Global climate change is resulting in long-term shifts in temperatures and weather patterns. This review highlights primary metabolic adjustments of plants to cope with future abiotic stressors.

## Introduction

**Fig. 1.**
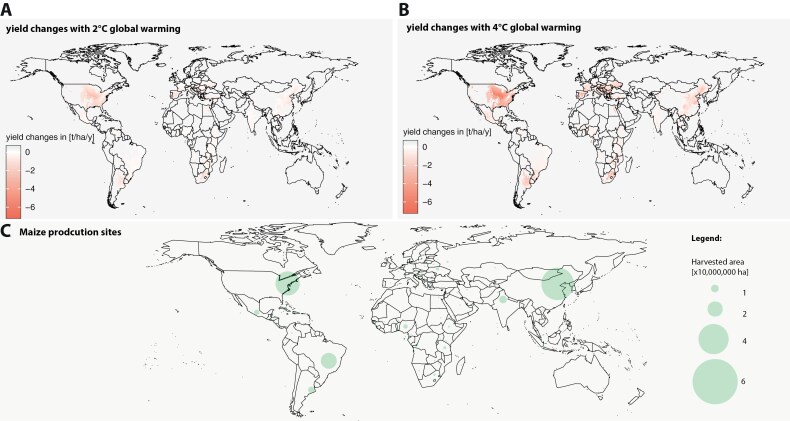
Impact of climate change on yield potential. (A) and (B) demonstrate the predicted effect of increased temperature by 2 °C and 4 °C, respectively, on yield (t) per hectare per year. The data were obtained from Tigchelaar *et al.* (2018). (C) Global harvested maize area country-wise (highlighted at the capital locations) with a factor of 10 000 000 ha for the year 2022. Size reflects the harvested area. The data was obtained from FAOSTAT.

One of the most pressing challenges for 21^st^ Century agriculture is ensuring food security for the growing population in the face of climate change. This is not only a case of ensuring crop yield but also crop quality since due to the sessile nature of plants, their metabolism needs to be highly plastic and as such is highly responsive to environmental changes (Fettke and Fernie, 2025). Photosynthetic organisms, and in particular plants, have played a pivotal role in shaping Earth’s biosphere and atmosphere (Fernie and Bauwe, 2020; Xu and Weng, 2020; Fernie *et al.*, 2024), the latter through the combined activities of oxygen production and carbon sequestration. In turn, the diversity of plants has been moulded by historical climatic changes leading to developmental and metabolic innovations (Fernie *et al.*, 2024). Whilst climate change has been occurring across millennia and is an ongoing process, it is widely acknowledged that this is now proceeding at a greatly accelerated rate due to anthropogenic activities in the post-industrial era (Parrenin *et al.*, 2013; Bereiter *et al.*, 2015; Xu and Weng, 2020). Atmospheric concentrations of CO_2_ which have remained remarkably stable in the range of 200–300 ppm over the past 800 000 years have been gradually increasing since the industrial revolution, recently passing a historic high of 400 ppm in 2015 and continuing to rise (Parrenin *et al.*, 2013; Xu and Weng, 2020). Indeed, the average annual increase which is currently in excess of 2 ppm may seem minor yet is two orders of magnitude higher than that recorded in the geographic record (Bereiter *et al.*, 2015; Royer, 2016). This elevation in atmospheric concentrations of CO_2_ is mirrored by enhanced global temperatures with the global average temperature increasing by 1.5 °C over the same period (Ourbak and Tubiana, 2017). In Fig. 1 we show the future climate impact of increasing temperature by 2 °C and 4 °C on yield changes, respectively [data from Tigchelaar *et al.* (2018)]. Damage to the stratospheric ozone layer has additionally led to an elevated intensity of ultraviolet (UV) radiation and ozone levels on the ground (Marenco *et al.*, 1994; Morgan *et al.*, 2003; United Nations Environment Programme, 2017). Climate change scenarios are also predicting both an increased frequency and increased affected global area experiencing extreme climates including massive increases in drought and salt stresses (Hassani *et al.*, 2021). Indeed, as stated by the PlantACT consortium (Hirt *et al.*, 2023), established to explore the possible contributions of plant science to mitigate against climate change, current focus of crop adaptation relates to resistance to drought, heat, salinity, and flooding with different regions needing crops to be adapted to different stressors (Hirt *et al.*, 2023). Naturally plants as carbon-fixing organisms can grow more robustly in the presence of high CO_2_ (Zhu *et al.*, 2016). Moreover, in the past they have tolerated far greater fluctuations than experienced in current atmospheric CO_2_ levels. Indeed, over the estimated 470 million years of plant evolution, when plants first transitioned to the terrestrial environment atmospheric CO_2_ levels were 10 times higher than present levels at around 400 ppm (Rothman, 2002). That said, plants may fail to cope with the associated elevations in temperature, increased incidence of drought and salt stress, and enhanced levels of UV and ground level ozone stress and their combinations. The rise of global ambient temperature threatens the growth and yield of major crops including rice which is extremely sensitive to high temperature from the seedling to the reproductive stage (Shi *et al.*, 2023) and it has been estimated that the world’s rice yield loss will be 40% over the next century due to the negative effects of high temperature (Jagadish *et al.*, 2015). Similarly, amongst stable crops rice is the most sensitive to salinity stress (Chen *et al.*, 2021); however, its effects reach far beyond rice since it now effects 62% of arable land worldwide, with in excess of 1 billion hectares being severely affected by salinity (Chen *et al.*, 2021). In a similar vein, in 2023 the drought-impacted area in the European Union was determined at 143 513 km^2^ (European Environment Agency, 2024). Furthermore, increased levels of crop UV-B exposure, as a result of ozone depletion has been projected to reduce global crop productivity by 20–25% (Fiscus and Booker, 1995), while yield reduction due to O_3_ pollution has been estimated to reduce wheat, rice, maize and soybean yield by 4–15%, 3–4%, 2–5%, and 5–15%, respectively (Van Dingenen *et al.*, 2009; Avnery *et al.*, 2011). In addition to changes in yield, many plants are already experiencing phenotypic changes as a consequence of rapid climate change. For example, important commercial crops such as coffee (*Coffea arabica* and *Coffea canephora*), tea (*Camellia sinensis*), sugarcane (*Saccharum* spp.), grape (*Vitis* spp.), rubber tree (*Hevea brasiliensis*), and oil palm (*Elaeis* spp.) have undergone extensive artificial selection to favour certain growth traits (Herms and Mattson, 1992; Chen *et al.*, 2015; Gaillard *et al.*, 2018). Other species, which are the sources of commercial materials such as mangroves (*Rhizophora* spp.), are found in niche environments and face habitat loss (Nilesh Lakshman *et al.*, 2019). Moreover, the taste qualities of both tea and coffee have been documented to have changed due to climate change (Ahmed *et al.*, 2014) and the continued viability of kava (*Piper methysticum*), which produces bioactive kavolactones, since kava plants which are highly sensitive to temperature, humidity, and soil composition, are at risk (Pluskal *et al.*, 2019). In recognition of the dramatic consequences of climate change on both crop yields and quality, considerable research has focused on the effects of changes in these parameters on plant physiology and metabolism. Given that three recent articles provide a very comprehensive evaluation of the effects of climate change on the levels of plant specialized metabolites (Xu and Weng, 2020; Sun *et al.*, 2023; Sun and Fernie, 2024) we will focus here on plant primary metabolism. For this purpose, we will review documented changes in photosynthesis, respiration and photorespiration, and plant growth as well as the levels of primary metabolites in plants exposed to: (i) elevated CO_2_; (ii) elevated temperature; (iii) water-deficit stress; (iv) salinity stress; (v) high light stress, and (vi) ground-level ozone stress. We will largely illustrate at a metabolite by metabolite level with studies in Arabidopsis given the richness of available data, before discussing more broadly with respect to recent data for crop species. Given that these stresses rarely occur in isolation but rather normally occur simultaneously or in concert with one another (Zhang and Sonnewald, 2017; Zandalinas *et al.*, 2021), we additionally discuss studies in which the effects of multi-factorial stresses are assessed at any of the above levels [reviewed in Bulut *et al.* (2025)].

### Hormonal regulation of plant responses to abiotic stress

**Fig. 2.**
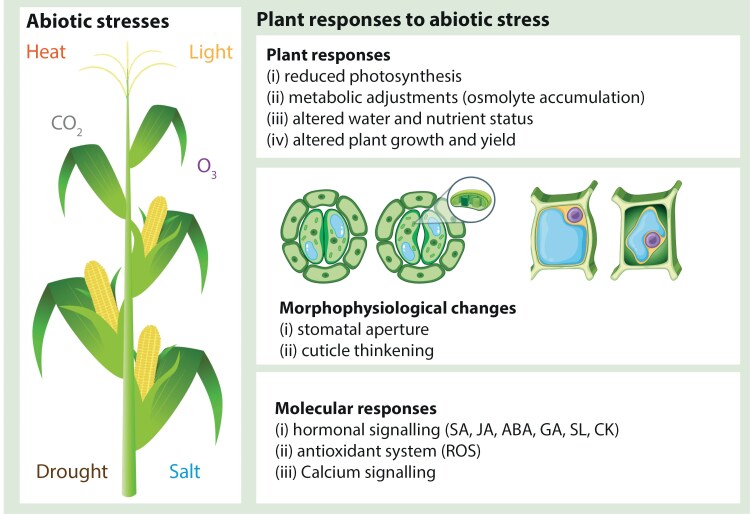
Schematic illustration of plant responses to abiotic stress. Common aspects of plant responses highlighting morphophysiological changes and molecular responses. SA = salicylic acid; JA = jasmonic acid; ABA = abscisic acid; GA = gibberellic acid; SL = strigolactone; CK = cytokinin.

Plant hormones orchestrate growth, development, and environmental stress responses through intricate signalling networks (Fig. 2). Abiotic stresses such as drought, salinity, and heat trigger adaptive mechanisms involving hormonal crosstalk. Calcium signalling plays a pivotal role in osmosensing, with rapid cytosolic Ca²⁺ fluctuations initiating downstream responses (Stephan *et al.*, 2016). Abscisic acid (ABA) is central to stress adaptation, with its biosynthesis regulated by NCED3 and modulated by transcription factors such as ATAF1 and NGATHA proteins (Sato *et al.*, 2018). ABA promotes stomatal closure through SnRK2 kinases, activating ion channels and integrating signals from CO₂, light, and other stimuli to optimize water use (Yoshida *et al.*, 2015).

ABA-responsive elements (ABREs) and AREB/ABF transcription factors mediate transcriptome remodelling, enabling plants to adjust metabolism and growth under stress (Marcotte *et al.*, 1989). The TOR pathway reciprocally regulates ABA signalling, balancing growth and stress responses (Wang *et al.*, 2018). For example, during seed germination, ABA and gibberellins (GAs) exert antagonistic control, with DELLA proteins modulating dormancy and germination. Environmental signals, such as light and temperature, influence this balance, with ICE1 inhibiting DELLA-ABI5 complexes to facilitate germination (Hu *et al.*, 2019).

In flowering regulation, ABA promotes drought escape by interacting with photoperiod-dependent regulators like GIGANTEA and SOC1, while salt stress delays flowering through ethylene accumulation and DELLA stabilization (Riboni *et al.*, 2016; Hwang *et al.*, 2019). ABA signalling extends to systemic stress responses, integrating hormonal pathways such as strigolactones, cytokinins, and jasmonates (Waters *et al.*, 2017). Under drought, ABA-induced stomatal closure conserves water, while in heat stress, jasmonates fine-tune stomatal dynamics for cooling. High CO₂ levels modulate stomatal responses independently of ABA, involving CBC kinases and Raf-like proteins (Hashimoto-Sugimoto *et al.*, 2016). Hormonal crosstalk fine-tunes responses to fluctuating environmental conditions, with ABA, cytokinins, and brassinosteroids coordinating developmental plasticity. Advances in understanding these interactions provide insights into improving stress resilience and optimizing plant performance under adverse conditions.

### Elevated CO_2_ levels

**Fig. 3.**
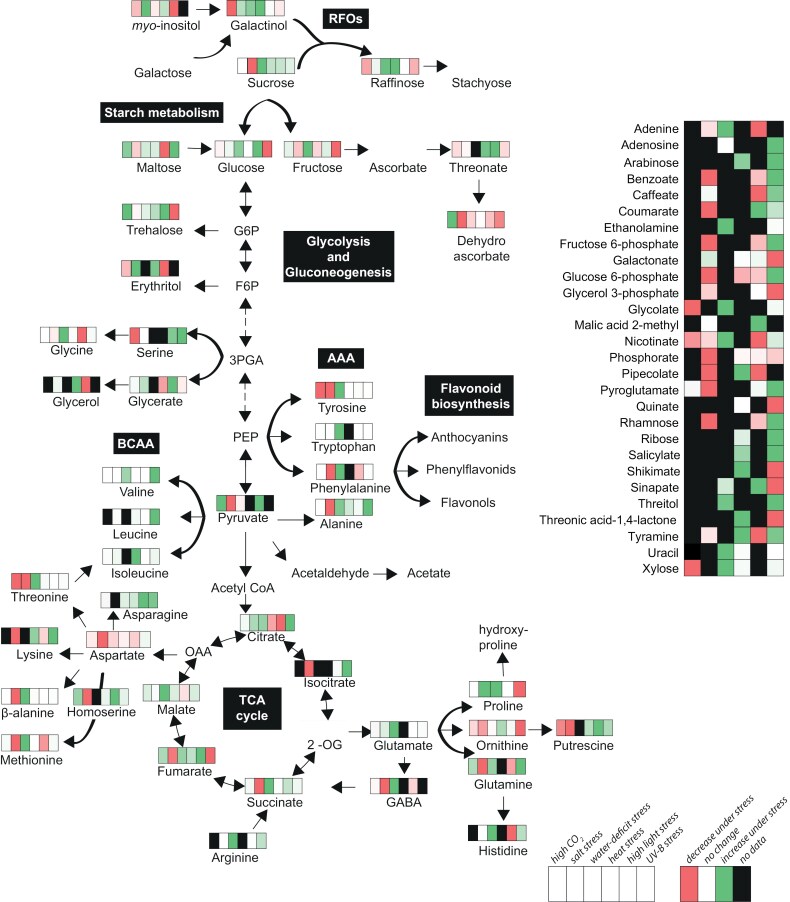
Metabolic response of wild-type Arabidopsis plants to several abiotic stresses, including high CO_2_, salt, water-deficit, heat, high light, and UV-B. Depicted data are extracted from Florian *et al.* (2014), Segarra-Medina *et al.* (2023), Urano *et al.* (2009), Kaplan *et al.* (2004), and Kusano *et al.* (2011). Colours depict the relative accumulation levels of each metabolite compared to controlled condition in each respective study. OAA = oxaloacetate, 2-OG = 2-oxoglutarate, G6P = glucose 6-phosphate, F6P = fructose 6-phosphate, PEP = phospho*enol*pyruvate, 3PGA = 3-phosphoglycerate, RFOs = raffinose family oligosaccharides, GABA = γ-aminobutyric acid, BCAA = branched chain amino acid, AAA = aromatic amino acid, and TCA = tricarboxylic acid.

As we already mentioned, elevated CO_2_ levels would generally be anticipated to result in elevated rates of photosynthesis and increased plant productivity (Ainsworth and Rogers, 2007). They would additionally be anticipated to result in lower rates of photorespiration and rates of respiration (Dusenge *et al.*, 2019). Given the high degree of interaction between these pathways, such changes could be anticipated to result in a major rewiring of plant primary metabolism. Fig. 3 shows the changes in metabolite levels when *Arabidopsis thaliana* plants are subjected to elevated CO_2_ concentrations of 155 Pa (Florian *et al.*, 2014). Intrinsically, the levels of sucrose, fructose, and maltose increased while the sugar alcohols galactinol, *myo*-inositol, and erythritol decreased, as did raffinose. Ascorbate and dehydroascorbate levels increased as did the phospho*eno*lpyruvate-derived amino acid tryptophan and the oxaloacetate (OAA) derivative homoserine. The tricarboxylic acid (TCA) cycle intermediates, namely 2-oxoglutarate, fumarate, malate, and succinate, respond with an increase, while the levels of putrescine, serine, and threonine all decreased. The tendency of metabolite levels to increase is likely simply the consequence of an enhanced carbon assimilation. In the crassulacean acid metabolism (CAM) bromeliad *Aechmea*, Ceusters *et al.* (2008) demonstrated that under elevated CO_2_ an increase of 60% of carbon gain was observed, linked largely to gains in the accumulation of hexose sugars. In another case, Noguchi *et al.* (2018) investigated the increase in atmospheric CO_2_ on respiration rates of rice cultivars. Among the measures, CO₂ efflux, O₂ uptake, nitrogen content, amount of primary metabolites, and respiratory enzyme activities were determined. Elevated CO₂ reduced leaf CO₂ efflux in both rice varieties but remained higher in high-yielding Takanari than in Koshihikari. Leaf O₂ uptake was largely unaffected by CO₂ or variety. Increased water temperature had no significant impact on CO₂ efflux or O₂ uptake. Takanari exhibited higher N and amino acid content, suggesting enhanced N assimilation, which may have increased respiratory NADH consumption and CO₂ efflux. In Koshihikari, elevated CO₂ altered TCA cycle intermediate ratios and reduced maximal enzyme activities, likely contributing to its lower CO₂ efflux rates. Aside from these metabolic changes, understanding how guard cells regulate stomatal aperture in response to CO₂ is crucial for developing climate-resilient crops and refining plant response models. Recent findings highlight CO₂ signalling pathways, secondary messengers, carbonic anhydrase localization, ABA interactions, and photosynthesis in stomatal responses. Additionally, insights into stomatal development and CO₂-mediated repression suggest new signalling models and research directions [reviewed in Engineer *et al.* (2016)].

It is, however, important to note that this is the effect of elevated CO_2_ in isolation and does not take into account the consequential effects on other parameters such as temperature and ozone that elevated CO_2_ levels provoke. Indeed, such changes render predictions as to its effect even on photosynthetic rates difficult to predict. The effect of elevated CO_2_ has been evaluated not just in the model plant Arabidopsis but also in a wide range of other species. Indeed, free air carbon-dioxide enrichment (FACE) experiments whereby the CO_2_ level of part of a field (or forest) are exposed to artificially elevated CO_2_ levels have been carried out for a wide range of plant species, such as soybean (Gillespie *et al.*, 2012). Moreover, several greenhouse-based trials have been carried out. In several crop species, including tomato, pepper, strawberry, and cucumber, it has been shown that increasing CO_2_ concentration inherently results in an increase in yield [reviewed in Doddrell *et al.* (2023)]. The increase in yield is simply explained by mass action with the greater photosynthetic substrate availability cascading through metabolism. Concurrent with the increase in yield, sugar levels are likewise elevated. By determining the optimal partial CO_2_ concentration, by which the rate limiting factor of RuBisCO is removed, crop cultivation can be improved for high yielding crops without compromising for flavour.

### Water-deficit stress

The metabolic response to drought stress has recently been reviewed (Fàbregas and Fernie, 2019; Yoshida and Fernie, 2024); however, given the importance of this stress within the context of climate change scenarios we thought it vital to also include it here. Water deficit normally triggers stomatal closure in order to offset transpiratory losses consequently resulting in reduced rates of photosynthetic carbon assimilation (Pinheiro and Chaves, 2011). It is additionally suggested to impact photorespiration with an increase in levels while the effect of water deficits on dark respiration remains unclear, with studies reporting decreases, stability, or even increases in this process (Gimeno *et al.*, 2010). Reduced respiration under drought conditions has been observed in actively growing roots and mature leaves of crops and herbaceous species (Ribas-Carbo *et al.*, 2005; Galmés *et al.*, 2007). This decline may be attributed to limited substrate availability for mitochondria due to reduced photosynthesis, as well as inhibited leaf growth, particularly affecting the growth component of respiration (Flexas *et al.*, 2006; Gimeno *et al.*, 2010). However, under severe water stress, an increased demand for respiratory ATP might be necessary to support photosynthesis repair mechanisms, compensating for decreased ATP production in chloroplasts, as proposed by Flexas *et al.* (2006) and Atkin and Macherel (2009). At the metabolic level water-deficit stress is very well studied as is the role of the phytohormone ABA—particularly in Arabidopsis. As Fig. 3 shows, sugars and sugar derivatives all increase, as do the TCA cycle intermediates and the vast majority of amino acids, adenine, ethanolamine, glycolate, nicotinate, sinapate, threitol, uracil, xylose, and threonine. Indeed, the only metabolites whose levels decreased under this stress were dehydroascorbate and aspartate.

Given its importance, water-deficit stress has also been characterized at the metabolic level in a wide range of crop species. In line with the reports in Arabidopsis, it was demonstrated that tryptophan levels enhance drought tolerance in maize, while being accumulated under water-deficit stress (Zhang *et al.*, 2021). In addition, several phenolamides, such as coumaroyl-putrescine, feruloyl-putrescine, feruloyl-tyramine, and *N*-acetyl-putrescine, as well as lipids were up-regulated. In terms of amino acid derivatives, polyphenols and several organic acids were up-regulated, while a small proportion was reduced. Depending on the ecotype, the responses may vary. Drought-tolerant lines accumulate sterols, and dipeptides, while sensitive lines accumulate simple sugars and polyunsaturated lipids (Yang *et al.*, 2018). In rice, drought-responsive metabolites were determined by performing an enrichment analysis, where hormones, vitamins, and amino acids and derivatives showcased the largest change in their levels (Guo *et al.*, 2024). For tomato, several studies have assessed metabolic changes in vegetative and reproductive tissue water-deficit stress conditions, one of which demonstrated changes in sugar compositional changes in apoplastic hexose:sucrose ratios (Garchery *et al.*, 2013). Further, it was demonstrated that l-threonic acid, fructose, and sugar alcohols are involved in the adaptive response to water-deficit conditions within the pod-filling stage of chickpea. The biochemical mechanisms governing trait variation revealed dynamic interactions among metabolic pathways across temporal stages, highlighting increased flux towards inositol interconversions, enhanced glycolytic activity in high-performing genotypes, fumarate-to-malate conversion, and perturbations in carbon and nitrogen metabolism (Chaturvedi *et al.*, 2024).

### Salinity stress

Global soil salinization is a major environmental and socioeconomic issue that will intensify in the face of climate change (Hassani *et al.*, 2021). In line with drought stress, salinity stress demonstrates disruption of plant water relations and osmotic balance, leading to cellular damage and impaired metabolic activities (Dinneny, 2015). Further, reactive oxygen species (ROS) production increases causing oxidative damage. Contrary to what is found in drought stress, imbalance in ion homeostasis caused by salinity stress disturbs nutrient uptake and transport resulting in impaired plant growth and performance (Obata and Fernie, 2012). Consequently, plants readjust their metabolism to cope with the osmolarity and ionic disorders caused by salinity stress (Gong *et al.*, 2005; Obata and Fernie, 2012). As demonstrated in Fig. 3, TCA cycle metabolites mostly decrease, while citrate accumulates. Furthermore, most amino acids, including methionine, lysine, alanine, glycine, and glutamine as well as aromatic amino acids (AAAs) are down-regulated under salinity stress. Contrary to this, proline, as reported in many studies and serving as an osmotic and ionic stress marker, increases (Obata and Fernie, 2012), as do the branched-chain amino acids (BCAAs). Sugars, such as fructose, maltose, trehalose, and sucrose decrease, while glucose and raffinose increase. In the case of sugar alcohols, most increase under salinity stress. These metabolic adjustments include the accumulation of compatible solutes such as sugars, alcohols, and amino acids such as proline, which help maintain osmotic balance and protect cellular structures (Munns and Tester, 2008; Munns and Gilliham, 2015; Batista‐Silva *et al.*, 2019). In parallel, plants activate antioxidant defence systems to scavenge ROS and minimize oxidative damage (Bose *et al.*, 2014). High salt concentrations adversely affect agricultural crop yields by reducing enzymatic activity and photosynthetic efficiency (Lee *et al.*, 2013). To date, metabolic investigations in multiple crops species have been performed under salinity stress including wheat (Guo *et al.*, 2015), where salt stress led to an increase in the levels of glucose, glucose 6-phosphate, fructose 6-phosphate, 3-phosphoglycerate (3-PGA), and phospho*enol*pyruvate (PEP), all key components of glycolysis. Additionally, levels of fructose, trehalose, proline, valine, isoleucine, and leucine also increased. In contrast, salt stress caused a decline in fumarate and malate, both involved in the TCA cycle, as well as in maltose, shikimate, and quinate, which are part of the shikimate pathway. For maize, it was demonstrated that application of amino acids, such as valine, threonine, isoleucine, and aspartate, enhanced plant fitness under salt-stressed conditions (Wu *et al.*, 2024). Similar to drought responses, in tomato associations of polyamine reallocation via transporters as well as uptake and its modifications have been demonstrated to enhance salt tolerance (Yang *et al.*, 2024). While utilizing other approaches, such as grafting, key responsive and correlated metabolites, including malate, citrate, and aspartate, were identified across the graft collection, with their variance partially attributed to rootstock origin, resulting in distinct set of six metabolites—sorbose, galactose, sucrose, fructose, *myo*-inositol, and proline—the presence of which consistently characterized exceptional graft performance (Song *et al.*, 2022).

### Heat stress

Temperature stress has a large impact on reproductive tissues and thus is potentially highly harmful to yield (Kan *et al.*, 2023). Increasing global temperature is arguably the poster-child of climate change, it certainly creates the most news headlines. Increasing global temperature has massive implications for agriculture given that it will result in a shift in the arable areas suitable for both tropical and temperate crops. Increased temperature is likely to result in reduced rates of photosynthesis, and increase in photorespiration (Long, 1991) and respiration (Seydel *et al.*, 2022), rendering it difficult to predict its impact on plant productivity.

Photosynthesis is essential for plant growth and biomass production, yet it is one of the most heat-sensitive physiological processes. Under heat stress (HS), RuBisCO activity is significantly reduced due to the heat instability of RuBisCO activase [RCA, Wang *et al.* (2010)]. The large RCA isoform supports photosynthetic acclimation under moderate HS, while the short isoform primarily maintains RuBisCO’s initial activity under normal conditions (Wang *et al.*, 2010).

In Arabidopsis, the impact of a 40 °C heat shock stress of 4 h was demonstrated to result in a complex suite of metabolic changes (Kaplan *et al.*, 2004). While sucrose, *myo*-inositol, galactinol, raffinose, trehalose, erythritol, and maltose all increased, glucose was unchanged and fructose decreased. Similarly, glycerate decreased but glycerol increased. Moreover, alanine and isoleucine increased. Regarding the TCA cycle intermediates, citrate increased while fumarate and malate increased. Similarly, ornithine, putrescine, and lysine increased as did arabinose, asparagine, homoserine, pipecolic acid, ribose, salicylic acid (SA), shikimate, 1,4-lactone-threonate, and tyramine. The vast majority of metabolite levels increased, decreased levels were only observed for aspartate, fructose, and citrate in keeping with the fact that temperature will generally accelerate enzymatic processes as described by the Arrhenius equation (Borghi *et al.*, 2019). Likewise, Serrano *et al.* (2019) demonstrated that the carbohydrates, namely sucrose and raffinose family oligosaccharides (RFOs), and lipid pathway metabolites exhibited the most significant increases. Under HS, a notable crosstalk emerges between carbohydrate metabolism, specifically, the thermomemory metabolites stachyose, galactinol, and raffinose, and tyrosine metabolism, facilitating the production of salidroside, a phenylethanoid glycoside involved in thermomemory. Additionally, interactions were demonstrated between two glycerophospholipid pathways, the biosynthetic pathway of the thermomemory metabolite S-adenosyl-l-homocysteine and the terpenoid backbone, and the δ-tocopherol (chloroplast lipid) pathway. These interactions promote the synthesis of glycine betaine and other essential tocopherols, both of which play a critical role in enhancing plant tolerance to abiotic stress (Serrano *et al.*, 2019).

Given its importance HS has also been well characterized at the metabolic level in a wide range of crop species. In soybean exposed to HS for 24 h, 15% of the heat-induced proteins were involved in carbon and carbohydrate metabolism, with peak gene expression occurring at 12 h (Ahsan *et al.*, 2010). Notably, proteins related to carbon assimilation and photosynthesis were down-regulated during this time (Ahsan *et al.*, 2010). In contrast, heat acclimation for 14 d at day/night temperatures of 30 °C/27 °C in wheat (*Triticum aestivum*), barley (*Hordeum vulgare*), and oat (*Avena sativa*) varieties led to a reduction in hexose and sucrose concentrations (Janda *et al.*, 2021), while in tomato, altering HS transcription factors resulted in accumulation of metabolites, positively associated with thermotolerance, namely sucrose, glucose, and putrescine (Paupière *et al.*, 2020). Likewise, heat-induced accumulation of sucrose and triacylglycerols was demonstrated in fruits (Almeida *et al.*, 2021). In maize, accumulation of soluble sugars, proline, and malonyldialdehyde was demonstrated for three different cultivars under HS (Liu *et al.*, 2022).

In considering how to cope with the increased temperature, we should be open to lessons from the history of agriculture since many of our crops have already undergone massive adaptations in terms of temperature albeit it almost exclusively in the tropical to temperate direction (Fernie and Yan, 2019; Bellucci *et al.*, 2021). That said, they do provide an immense and comprehensive record regarding adaptation to temperature which will certainly be of a great benefit in climate-change oriented breeding.

### High light stress

The depletion of the ozone layer by the release of chloro-fluoro-carbons (CFCs) has been largely mitigated by their subsequent ban in the Montreal Protocol (Young *et al.*, 2021). That said, there are still areas of the globe where the ozone layer is thinner and crops are subjected to high light stress (United Nations Environment Programme, 2017). We differentiate here between high light stress and UV-stress despite the fact that they often occur simultaneously. High light stress can constrain photosynthetic rates due to photoinhibition, it is additionally reported to result in higher rates of photorespiration and respiration (Fu and Walker, 2023).

Fig. 3 documents the changes in primary metabolites following exposure to a light intensity of 650 μmol m^−2^ s^−1^ for 7 h [data from Segarra-Medina *et al.* (2023)]. High light stress resulted in mixed changes with regards to the levels of sugars and their derivatives with *myo*-inositol, maltose, and erythritol increasing, whereas sucrose, fructose, glucose, and trehalose decreased. Dehydroascorbate also decreased significantly, whereas serine and glycerate increased, whilst glycine and glycerol decreased. Following Cross-over Theorem (Newsholme and Start, 1973) this implies an inhibition of the reactions linking serine to glycine and glycerate to glycerol, respectively. High light stress also resulted in an increase of pyruvate and a decrease in phenylalanine. As for the other conditions described above, the TCA cycle intermediates displayed converse responses with the levels of citrate and malate decreasing while those of succinate and fumarate increased. In addition, lysine, methionine, histidine, glutamine, γ-aminobutyric acid (GABA), and dehydroascorbate decreased while putrescine and threonate increased . In a recent genome-wide study, it was shown that ascorbate levels are regulated under high light acclimation through interaction of PAS/LOV PROTEIN with VITAMIN C DEFECTIVE 2 [VTC2; Aarabi *et al.* (2023)]. Under high light conditions, plants enhance NADPH-consuming CO₂ assimilation, leading to increased accumulation of starch, C4 acids, such as fumarate and malate, and isoprenoids. The Calvin–Benson–Bassham (CBB) cycle and photorespiration, interconnected through RuBisCO’s oxygenase activity, result in the transient accumulation of the toxic intermediate 2-phosphoglycolate (Balcke *et al.*, 2024).

In addition to these changes, adenine, aspartate, benzoate, caffeate, fructose 6-phosphate, glucose 6-phospate, nicotinate, pipecolate, rhamnose, and tyramine decreased while asparagine, coumarate, galactonate, glycerol 3-phosphate, homoserine, 2-methyl-malate, pyroglutamate, and sinapate increased.

### UV stress

Sunlight inevitably also results in exposure to UV light, with particular importance given to the 280–320 nm wavelength (UV-B). This wavelength is a source of potential damage including to DNA with additional increase of free radical production. The threat is likely to be exacerbated by the progressive thinning of the ozone layer (United Nations Environment Programme, 2017). In order to counteract this threat, plants have evolved various morphological and molecular ways to adapt. Among others, focus here is given to metabolic adjustments to: (i) produce UV-B scavenging compounds and (ii) increase antioxidant compound production to maintain metabolic homeostasis. Compounds with UV-B scavenging properties belong mostly to the chemical superclass ‘phenylpropanoids and polyketides’, and will not be discussed here in detail, as they have been extensively reviewed elsewhere (Tohge and Fernie, 2017).

Sugars and sugar derivatives demonstrate no clear trends in altering levels under UV-B stress. Sucrose levels increase, while glucose and fructose are strongly reduced. In contrast, the levels of the starch degradation product maltose increase dramatically. Levels of amino acids also display relatively large changes. The 2-oxoglutarate-derived amino acids, glutamine and histidine increase, while ornithine and proline levels decrease. Oxaloacetate- and pyruvate-derived amino acids are likewise altered. Lysine, asparagine, aspartate, and homoserine dramatically increase. Similarly, the BCAAs all increase. Based on the time series of UV-B stress exposure, according to Kusano *et al.* (2011), biphasic responses are observed for certain primary metabolic pathways depending on changing the cellular context. The biphasic response to UV-B stress is marked by significant changes in primary metabolite levels, including ascorbate derivatives, and transcriptional regulation of genes involved in sugar synthesis (down-regulated) and energy production, as well as carbon flux toward the shikimate pathway (up-regulated). This aligns with the increased accumulation of specialized metabolites within the chemical superclass ‘phenylpropanoids and polyketides’, including elevated levels of aromatic amino acids (AAAs) under UV-B stress. It is well established that plants accumulate various phytochemicals, such as ascorbate, flavonoids, and sinapoyl malate, as a defence mechanism to mitigate the harmful cellular effects of UV-B irradiation (Chappell and Hahlbrock, 1984; Li *et al.*, 1993; Conklin *et al.*, 1996). This suggests that these changes result from altered carbon partitioning into and within the phenylpropanoid pathway, which can account for up to 30% of carbon flux in plants (Razal *et al.*, 1996; Huang *et al.*, 2010). As an aside, given their importance, we cover phenylpropanoids in this section. It was shown previously that Arabidopsis ecotypes adapted to high altitudinal and near equatorial environments produce saiginols, phenylacylated flavonoids with higher UV-B absorption than their precursor molecule (Tohge *et al.*, 2016). Regarding crop species, several (specialized) metabolites have been identified in potentially improving a plant’s resilience to UV-B stress, including rice (Peng *et al.*, 2017; Zhang *et al.*, 2024), qingke (Zeng *et al.*, 2020), wheat (Chen *et al.*, 2020), and tartary buckwheat (Huang *et al.*, 2024).

Reduced levels of these sugars may lead to the down-regulation of genes involved in cell wall and starch synthesis (Müller-Röber *et al.*, 1992) and lipid metabolism (Koch, 1996). In contrast, changes in the expression of genes related to TCA cycle intermediates, nucleotide metabolism, and amino acid biosynthesis only partially align with the steady-state levels of metabolic intermediates associated with these pathways. This suggests that substantial regulation occurs at multiple levels of metabolic control.

These findings have critical implications for global food security, as the exposure of crop plants to UV-B radiation has increased significantly in recent years, primarily due to the thinning of the ozone layer (Callaghan *et al.*, 2004; Isaksen *et al.*, 2009). Understanding the cellular carbon economy is vital for modelling plant responses and engineering crops that are better adapted to current and future environmental challenges.

### Low level ozone

Ozone (O₃) at the Earth’s surface is a highly reactive, short-lived air pollutant that poses significant risks to crop productivity. It affects crops directly by causing oxidative damage to cellular structures and indirectly by acting as a greenhouse gas, contributing to global warming. Since the Industrial Revolution, surface O₃ concentrations have more than doubled (Monks *et al.*, 2015). Although global levels vary geographically, many key agricultural regions are exposed to elevated O₃ levels during the growing season. Crops face two types of O₃ stress: chronic exposure to consistently high background levels throughout the season and acute stress when concentrations exceed approximately 100 parts per billion (ppb), triggering a hypersensitive response and promoting cell death.

Our understanding of ozone (O₃) uptake, perception, and cellular responses in field-grown plants under elevated [O₃] and fluctuating environmental conditions remains incomplete. However, evidence suggests the involvement of several known mechanisms. For instance, O₃-tolerant aspen clones limit hydrogen peroxide accumulation to the apoplast when exposed to chronically elevated [O₃] (~55 ppb). In contrast, O₃-sensitive clones accumulate hydrogen peroxide in the cytoplasm, leading to chloroplast damage and a reduction in peroxisome numbers under similar conditions (Oksanen *et al.*, 2004). In soybeans, chronic exposure to elevated [O₃] in field conditions increases the expression of genes involved in redox and antioxidant metabolism, as well as the TCA cycle and mitochondrial electron transport chain, likely to meet the heightened energy demands for detoxification (Gillespie *et al.*, 2012). Elevated ozone [O₃] levels disrupt primary metabolism, leading to reduced plant growth rates, smaller total leaf area, and decreased above- and below-ground biomass (Morgan *et al.*, 2003; Ainsworth, 2008; Feng *et al.*, 2008). However, the detailed mechanisms by which this occurs remain unestablished and there is even a paucity of metabolite measurements under these conditions.

These impacts on crop physiological processes accumulate over the growing season. For instance, the reduction in photosynthesis due to elevated [O₃] is more pronounced during reproductive stages than in vegetative stages. This is partly because leaves that develop late in the reproductive phase remain at the top of the canopy, where they are exposed to elevated [O₃] for extended periods (Morgan *et al.*, 2003; Feng *et al.*, 2008).

### Common responses

Metabolic responses to a multitude of abiotic stresses can vary. However, considering the primary metabolic data across the addressed stresses highlights a consistent increase of the BCAAs valine, leucine, and isoleucine, followed by arginine and asparagine as well as the polyamines. BCAAs have recently emerged as important stress-related metabolites—probably as the result of increased protein turnover under stress (Araújo *et al.*, 2011) functioning as important alternative energy sources to sustain mitochondrial respiration (Ishizaki *et al.*, 2005; Araújo *et al.*, 2011).

BCAAs, characterized by their short, branched hydrophobic side chains, accumulate significantly in plants under drought stress (Urano *et al.*, 2009; Joshi *et al.*, 2010). Similar to proline, BCAAs may contribute to improved drought tolerance by functioning as compatible osmolytes or serving as alternative energy sources (Huang *et al.*, 2010; Joshi *et al.*, 2010; Fàbregas and Fernie, 2019).

Joshi *et al.* (2010) proposed that BCAAs function as compatible osmolytes, as they exhibit significant accumulation under drought stress in various plant tissues. Another potential role of BCAAs during stress is their function as alternative electron donors for the mitochondrial electron transport chain (ETC). Typically, the ETC is fuelled by electrons from NADH and succinate to generate ATP. However, an alternative pathway exists, allowing electrons from other substrates to enter the ETC via the electron transfer flavoprotein (ETF) complex. Recent studies have underscored the significance of this alternative pathway under dark and stress conditions, particularly during carbon starvation (Ishizaki *et al.*, 2005).

A ^13^C-feeding experiment demonstrated that lysine and BCAAs are converted into d-2-hydroxyglutarate and isovaleryl-CoA *in vivo*, which serve as direct electron donors for the ETF complex through the action of d-2-hydroxyglutarate dehydrogenase and isovaleryl-CoA dehydrogenase (Araújo *et al.*, 2010). Thus, BCAAs supply electrons to the ETC both directly via the ETF complex and indirectly, as their catabolic products enter the TCA cycle (Araújo *et al.*, 2011). The primary source of accumulated BCAAs is likely protein degradation, which has recently been identified as an important alternative respiratory substrate under stress (Araújo *et al.*, 2011). Additionally, BCAA synthesis is up-regulated under drought stress (Urano *et al.*, 2009; Joshi *et al.*, 2010). These findings highlight the central role of BCAA metabolism in plant responses to abiotic stress conditions.

### Combinatorial stresses

As stated above, abiotic stresses are major challenges arising in the ongoing climate crisis. Despite this fact, the majority of studies performed to date are based on single stress experiments, which only represents frictions on the impact of climate change. In a recent review, we address the aspect of combinatorial stress on plant metabolism and demonstrate that, against previous reports, for given stress combinations, additive effect on metabolite levels can be achieved in model as well as crop species [reviewed in Bulut *et al.* (2025)]. Further, an intriguing finding is that, despite the common assertion that plant responses to multifactorial stresses are generally neither additive nor predictable—claims often based on end-phenotypes or, more frequently, transcriptomic data—metabolite-level changes appear to be both additive and predictable. This could be attributed to the overarching regulatory logic of metabolism. Fernie and Stitt (2012) explored this in detail, so we will only briefly note that this lack of predictability may stem from the inherent redundancy in plant metabolic networks, which likely buffer against the substantial shifts observed at the transcriptional level. A consequence of this is that to understand metabolic regulation measurements at the level of the metabolites themselves appears to be strictly necessary.

## Conclusion and Future Perspectives

In summary, research to date has catalogued an extensive range of metabolic changes in response to stress. While some of these changes are well understood at a mechanistic level, our knowledge of their causes and effects remains fragmented in certain areas.

Stress-induced metabolic changes can be broadly categorized into three phases: (i) the immediate impact of environmental changes, (ii) transient adaptation to stress conditions, and (iii) the establishment of a new steady state under prolonged stress. The duration of each phase varies depending on the type and severity of the stress.

To accurately link metabolic changes to specific phases, detailed time-course experiments, such as those conducted by Caldana *et al.* (2011) and Jozefczuk *et al.* (2010) are essential. Additionally, considering climate change, the interplay of several stresses needs to be further investigated to optimize plant metabolic and systemic reprogramming to thrive under the predicted global climate scenarios. Recently, as a result of natural genetic variation studies under stress conditions, namely salt and drought, applications of detected compounds have been found to increase plant resilience and fitness by improving yield outcome (Zhang *et al.*, 2021; Wu *et al.*, 2024). In addition, ecological niches can be altered as a result of climate change, making pest infestations more frequent and severe, which needs to be considered in the equation of improving overall plant resilience (Subedi *et al.*, 2023).

Furthermore, combining existing research with isotope labelling, phytohormone profiling, transcriptomics, proteomics, and phenomics will offer deeper insights into the molecular basis of adaptions to variable environments. Ultimately, this knowledge should serve as a robust foundation for metabolic engineering and synthetic biology approaches aimed at enhancing crop resilience. However, given the urgency of the problem we should realize that such experiments are crucial now and that initiatives such as PlantACT (Hirt *et al.*, 2023) are imperative if we are to ensure future global food security.
